# Supplementary material for: Surveillance of Erythrovirus B19 (B19V) in patients with acute febrile illness suspected of arboviruses in Mato Grosso do Sul state, Brazil
Source: Front Microbiol. 2024 Jul 18;15:1417434. doi: 10.3389/fmicb.2024.1417434 (PMC11291312; doi:10.3389/fmicb.2024.1417434)

Supplementary Material

Surveillance of Erythrovirus B19 (B19V) in patients with acute febrile illness suspected of arboviruses in Mato Grosso do Sul, Brazil

Gislene Garcia C. Lichs[1,5], Zoraida del Carmen Fernandez Grillo[2], Valdinete Alves do Nascimento[3], Daniel Maximo Corrêa Alcantara[2], Everton Ferreira Lemos[4], Cristiano M. Espínola Carvalho[6], Luiz Henrique Ferraz Demarchi[1], Crhistinne Carvalho Maymone Gonçalves[5,7], Felipe Gomes Naveca[3,8], Alexsandra Rodrigues de Mendonça Favacho[2,5]

1. **SES-MS—Laboratório Central de Saúde Pública de Mato Grosso do Sul, Campo Grande, Brazil**
2. **Fundação Oswaldo Cruz, Fiocruz Mato Grosso do Sul, Campo Grande, Mato Grosso do Sul, Brazil**
3. **Núcleo de Vigilância de Vírus Emergentes, Reemergentes ou Negligenciados, Instituto Leônidas e Maria Deane, Fiocruz, Manaus, Amazonas, Brasil**
4. **Universidade Estadual de Mato Grosso do Sul - UEMS, Campo Grande, Mato Grosso do Sul, Brazil**
5. **Postgraduate program in infectious and parasitic diseases, Universidade Federal de Mato Grosso do Sul, Campo Grande, Brazil**
6. **Universidade Católica Dom Bosco, Campo Grande, Mato Grosso do Sul, Brazil**
7. **Secretaria de Estado de Saúde de Mato Grosso do Sul, Campo Grande, Brazil;**
8. **Laboratório de Arbovírus e Vírus Hemorrágicos, Instituto Oswaldo Cruz, Fiocruz, Rio de Janeiro, Brazil**
9.

Captions of the figures:

Figure S1. Age group and gender of sampled patients. n, number of patients sampled by age group.

Figure S2. The B19V samples that underwent sequencing originate from different municipalities in different geographic areas, demonstrating the wide dissemination of the virus throughout the state.

Figure S1. Age group and gender of sampled patients. n, number of patients sampled by age group.

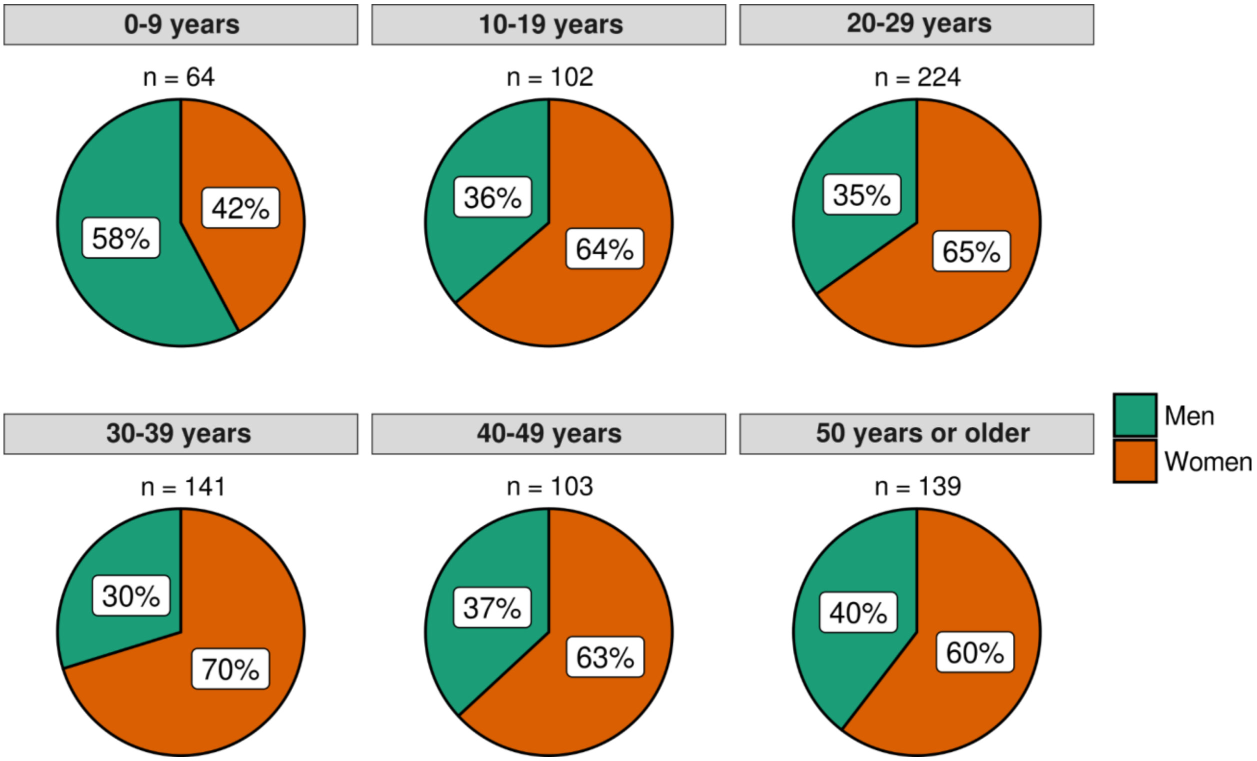

Figure S2. The B19V samples that underwent sequencing originate from different municipalities in different geographic areas, demonstrating the wide dissemination of the virus throughout the state.

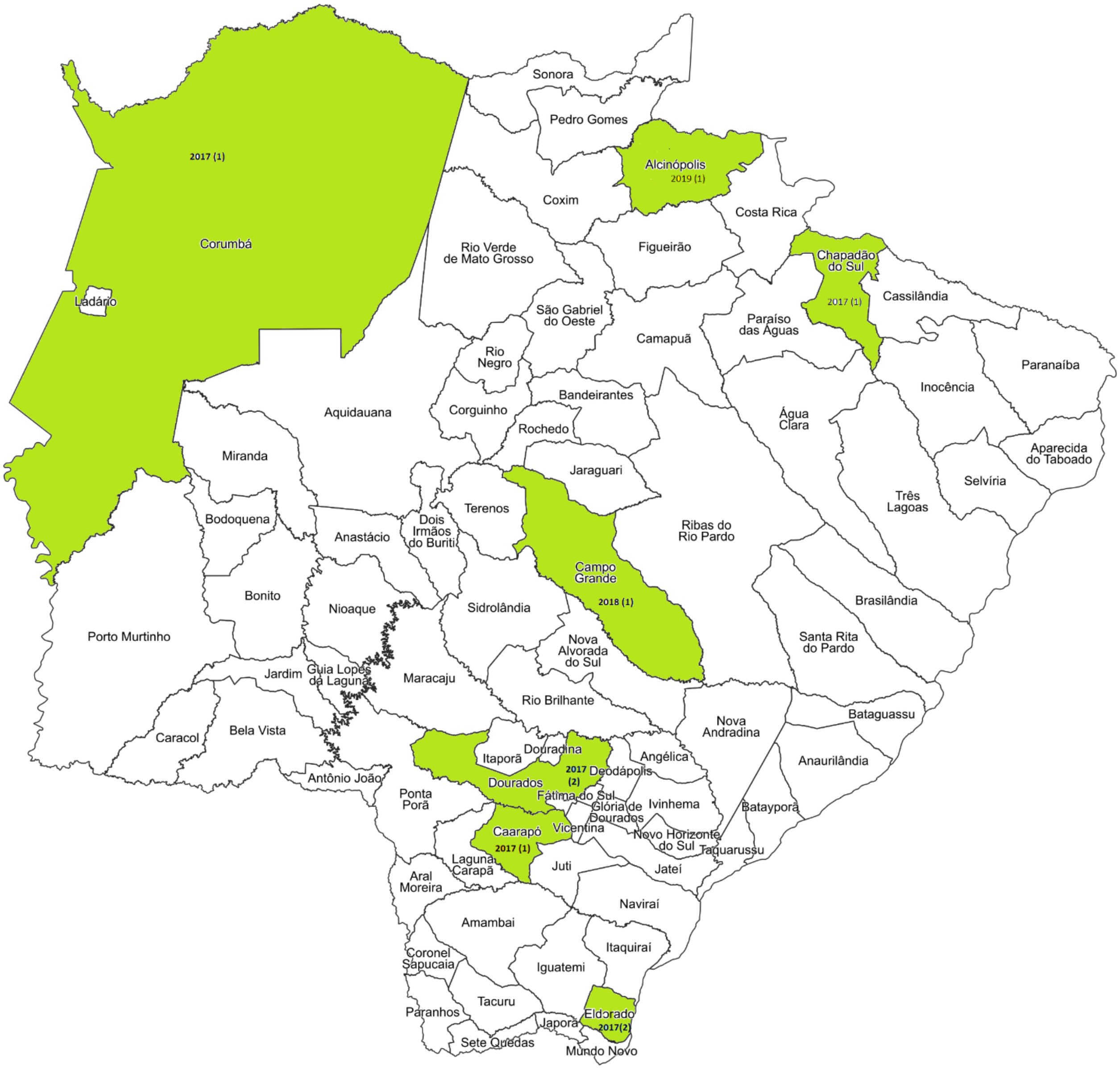

Supplement: Supplementary file 1 [file Data_Sheet_1.pdf]
