# Supplementary material for: Surveillance of Erythrovirus B19 (B19V) in patients with acute febrile illness suspected of arboviruses in Mato Grosso do Sul state, Brazil
Source: Front Microbiol. 2024 Jul 18;15:1417434. doi: 10.3389/fmicb.2024.1417434 (PMC11291312; doi:10.3389/fmicb.2024.1417434)
Supplement: Supplementary file 2 [file Data_Sheet_2.pdf]

Supplementary Material

Surveillance of Erythrovirus B19 (B19V) in patients with acute febrile illness suspected of arboviruses in Mato Grosso do Sul, Brazil

Gislene Garcia C. Lichs[1,5], Zoraida del Carmen Fernandez Grillo[2], Valdinete Alves do Nascimento[3], Daniel Maximo Corrêa Alcantara[2], Everton Ferreira Lemos[4], Cristiano M. Espínola Carvalho[6], Luiz Henrique Ferraz Demarchi[1], Crhistinne Carvalho Maymone Gonçalves[5,7], Felipe Gomes Naveca[3,8], Alexsandra Rodrigues de Mendonça Favacho[2,5]

1. **SES-MS—Laboratório Central de Saúde Pública de Mato Grosso do Sul, Campo Grande, Brazil**
2. **Fundação Oswaldo Cruz, Fiocruz Mato Grosso do Sul, Campo Grande, Mato Grosso do Sul, Brazil**
3. **Núcleo de Vigilância de Vírus Emergentes, Reemergentes ou Negligenciados, Instituto Leônidas e Maria Deane, Fiocruz, Manaus, Amazonas, Brasil**
4. **Universidade Estadual de Mato Grosso do Sul - UEMS, Campo Grande, Mato Grosso do Sul, Brazil**
5. **Postgraduate program in infectious and parasitic diseases, Universidade Federal de Mato Grosso do Sul, Campo Grande, Brazil**
6. **Universidade Católica Dom Bosco, Campo Grande, Mato Grosso do Sul, Brazil**
7. **Secretaria de Estado de Saúde de Mato Grosso do Sul, Campo Grande, Brazil;**
8. **Laboratório de Arbovírus e Vírus Hemorrágicos, Instituto Oswaldo Cruz, Fiocruz, Rio de Janeiro, Brazil**

Captions of the tables:

Table S1. Primers and probes were used in the study for B19V detection (NAVECA et al., unpublished data).

Table S2. Components and volumes used to prepare the reactions for B19V detection (NAVECA et al., unpublished data).

Table S3. Primers and probes are used for amplification of the entire genome of B19V.

Table S4. Results for 2017 data, with comparison for all 10 orientation models implemented in the R package 'CircMLE'.

Table S5. Results for 2018 data, with comparison for all 10 orientation models implemented in the R package 'CircMLE'.

Table S6. Results for 2019 data, with comparison for all 10 orientation models implemented in the R package 'CircMLE'.

Table S1. Primers and probes used in the study for B19V detection (NAVECA et al., unpublished data).

Oligonucleotides	Sequence
B19_FNF	5’ ACAAGCCTGGGCAAGTTAGC 3’
B19_FNR	5’ CATTRCCAGGCCCAACAT 3’
B19_FN_P	5’ (FAM) TACAACTACCCGGTACTAAC 3’

Table S2. Components and volumes used to prepare the reactions for B19V detection (NAVECA et al., unpublished data).

Component	10 µl reaction
Nuclease-free water	1.4 µL
2X PCR Master Mix Kapa Probe	5 µL
Primer mix (5 µM)	0.6 µL
Probe (10µM) FAM	0.1 µL
Rox Dye	0.4 µL
Template DNA	2.5 µL

Table S3. Primers used for amplification of near-to-complete B19V genome.

Name	Sequence 5’- 3’	Length (bp)
B19VGI_350FNF (forward)	CGGCATCTGATTTGGTGTCTTC	2037
B19VGI_2387FNR (reverse)	CACACATAATCAACCCCAACTAACA	
B19VGI_1605FNF (forward)	AAGTACAGGAAAAACAAACTTGGCAA	2258
B19VGI_3862FNR (reverse)	GTAAGCATATTGAGGGGGAAAGTATAC	
B19VGI_3298FNF (forward)	CCCAAGCATGACTTCAGTTAATTCT	1679
B19VGI_4976FNR (reverse)	GGAGTGTTTACAATGGGTGCAC	
B19VGI_4453F (forward)	GTTACAGGGTTTAAACATGCACAC	1144
B19VGI_5596R (reverse)	CCAAATCAGATGCCGCCG	

Table S4. Results for 2017 data, with comparison for all 10 orientation models implemented in the R package 'CircMLE'. The models are described by five parameters: the mean direction (φ1, in degrees) and concentration parameter (*k*1) for the first mode, the mean direction (φ2, in degrees) and concentration parameter (*k*2) for the second mode, and the proportional size of the first distribution (λ; the second distribution is thus fixed at size 1−λ). They were classified based on Akaike's Information Criterion with small-sample correction (AICc). ΔAICc, delta AICc; AICc w_i, model weights; ER, evidence ratio.

**Modelo**	**ϕ1**	***k*1**	**λ**	**ϕ2**	***k*2**	**AICc**	**ΔAICc**	**AICc w_i**	**ER**
M3A	4.40	3.18	0.50	7.54	3.18	59.11	0.00	0.38	-
M4A	4.39	3.20	0.65	7.53	3.20	60.68	1.58	0.17	2.20
M2C	4.66	49.99	0.25	-	0.00	61.27	2.17	0.13	2.96
M2B	4.50	5.80	0.50	-	0.00	61.60	2.50	0.11	3.49
M3B	4.45	4.05	0.50	7.59	1.64	61.81	2.70	0.10	3.86
M1	-	0.00	1.00	-	0.00	62.49	3.38	0.07	5.43
M4B	1.25	3.02	0.36	4.39	3.29	64.16	5.06	0.03	12.53
M2A	4.25	0.51	1.00	-	0.00	65.23	6.13	0.02	21.40
M5B	4.36	3.31	0.64	1.31	3.02	68.20	9.10	0.00	94.38
M5A	3.45	5.00	0.41	2.66	5.00	2000000011.33	1999999952.23	0.00	-

Table S5. Results for 2018 data, with comparison for all 10 orientation models implemented in the R package 'CircMLE'. The models are described by five parameters: the mean direction (φ1, in degrees) and concentration parameter (*k*1) for the first mode, the mean direction (φ2, in degrees) and concentration parameter (*k*2) for the second mode, and the proportional size of the first distribution (λ; the second distribution is thus fixed at size 1−λ). They were classified based on Akaike's Information Criterion with small-sample correction (AICc). ΔAICc, delta AICc; AICc w_i, model weights; ER, evidence ratio.

**Modelo**	**ϕ1**	***k*1**	**λ**	**ϕ2**	***k*2**	**AICc**	**ΔAICc**	**AICc w_i**	**ER**
M5A	5.08	4.62	0.47	2.49	4.62	71.17	0.00	0.38	-
M3A	5.37	3.63	0.50	8.51	3.63	71.44	0.27	0.33	1.15
M4A	2.23	3.64	0.54	5.37	3.64	74.04	2.87	0.09	4.20
M3B	2.26	4.48	0.50	5.40	2.84	74.05	2.88	0.09	4.22
M5B	2.49	4.49	0.54	5.09	4.82	74.56	3.39	0.07	5.45
M4B	2.25	4.10	0.53	5.39	3.15	77.03	5.86	0.02	18.73
M2C	2.12	30.86	0.25	-	0.00	79.63	8.46	0.01	68.53
M2B	2.26	16.46	0.50	-	0.00	80.36	9.19	0.00	98.94
M1	-	0.00	1.00	-	0.00	80.87	9.70	0.00	127.51
M2A	3.55	0.51	1.00	-	0.00	82.80	11.63	0.00	335.68

Table S6. Results for 2019 data, with comparison for all 10 orientation models implemented in the R package 'CircMLE'. The models are described by five parameters: the mean direction (φ1, in degrees) and concentration parameter (*k*1) for the first mode, the mean direction (φ2, in degrees) and concentration parameter (*k*2) for the second mode, and the proportional size of the first distribution (λ; the second distribution is thus fixed at size 1−λ). They were classified based on Akaike's Information Criterion with small-sample correction (AICc). ΔAICc, delta AICc; AICc w_i, model weights; ER, evidence ratio.

Modelo	ϕ1	*k*1	λ	ϕ2	*k*2	AICc	ΔAICc	AICc w_i	ER
M2A	1.31	1.46	1.00	-	0.00	77.34	0.00	0.72	-
M5A	0.33	2.58	0.37	1.82	2.58	81.43	4.09	0.09	7.73
M2C	1.33	2.15	0.75	-	0.00	81.65	4.32	0.08	8.66
M2B	1.39	2.36	0.50	-	0.00	82.11	4.78	0.07	10.89
M3B	4.62	0.00	0.50	7.76	2.24	84.84	7.51	0.02	42.64
M4B	4.40	0.00	0.27	7.54	2.30	85.00	7.67	0.02	46.20
M5B	6.28	2.53	0.49	1.71	3.82	88.08	10.74	0.00	215.33
M1	-	0.00	1.00	-	0.00	91.89	14.56	0.00	1450.15
M3A	1.52	0.98	0.50	4.66	0.98	95.82	18.48	0.00	10315.06
M4A	5.56	0.81	0.25	8.70	0.81	96.28	18.95	0.00	13008.91
